# Application of Pandemic Intelligence in Dynamic Data in Taiwan

**DOI:** 10.3390/ijerph18189925

**Published:** 2021-09-21

**Authors:** Tzu-Yin Chang, Wen-Ray Su, Hongey Chen, Ming-Wey Huang, Lu-Yen A. Chen

**Affiliations:** 1 National Science and Technology Center for Disaster Reduction, New Taipei 23143, Taiwan; wrsu@ncdr.nat.gov.tw (W.-R.S.); hchen@ncdr.nat.gov.tw (H.C.); mwhuang@ncdr.nat.gov.tw (M.-W.H.); 2 Institute of Clinical Nursing, School of Nursing, National Yang Ming Chiao Tung University, Taipei 11221, Taiwan; annychen@nycu.edu.tw

**Keywords:** COVID-19, data integration, dynamic intelligence, pandemic management

## Abstract

Taiwan was successful in containing the spread of the novel coronavirus (COVID-19) in 2020. One major factor in this success was the compilation and provision of comprehensive information about the pandemic. The present study proposes a pandemic intelligence system that provides data on the number of epidemic prevention professionals in each county and city, as well as daily confirmed cases, the demographics of the confirmed cases, and available resources (negative-pressure room beds and artificial ventilation apparatuses) in hospitals. Furthermore, the system provides the location of pharmacies selling masks and their current inventories, as well as the distribution of crowds at popular tourist destinations and social-distance monitoring. The most frequently used map layer in the thematic map of the pandemic is that of crowd distribution during the study period from March 2020 until the end of the same year. The case study used in this investigation for applying the system is represented by the 4-day weekend for Tomb-Sweeping Day of 2020. Through the real-time analysis of dynamic data and the integration of intelligence, the system offers a clear insight into changes in relevant information and, thus, enables the preemptive deployment of control measures by the county/city governments regarding pandemic management.

## 1. Introduction

**Figure 1:**
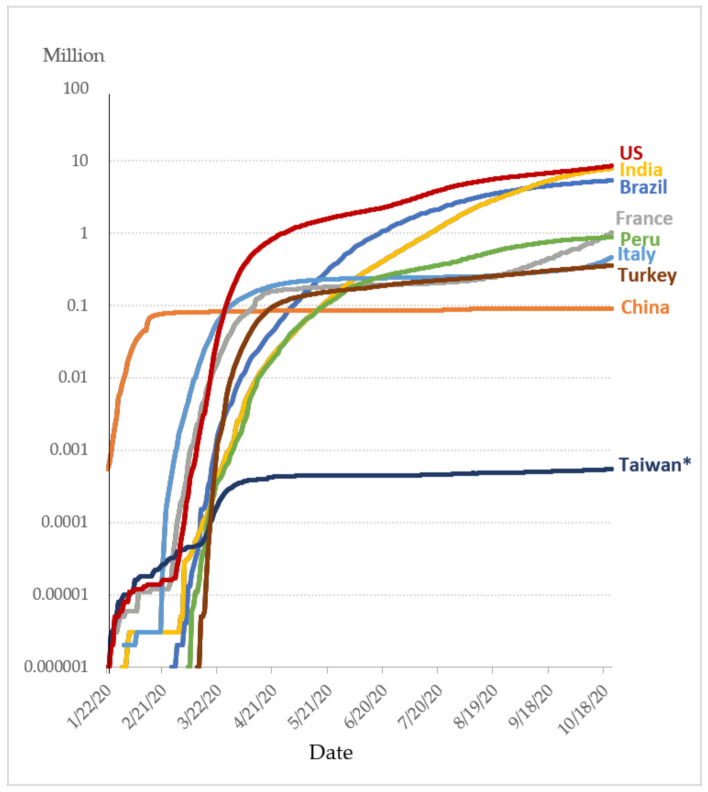
Confirmed COVID-19 cases in major countries (source: CSSE JHU, 2020) [2,7].

In early 2020, the novel coronavirus, severe acute respiratory syndrome coronavirus 2 (SARS-CoV-2) causing coronavirus disease (COVID-19; previously 2019-nCoV) spread rapidly in various countries. Data on the development of the pandemic (Figure 1) demonstrate that, prior to the end of February 2020, China was the only country with a high number of confirmed cases; however, beginning in March, large numbers of confirmed cases began to appear in other countries around the world [1]. Taiwan, too, began to see a rise in confirmed cases and deaths in March and, in response, enacted border controls.

Dong et al. (2020) established a COVID-19 dashboard for collecting and providing dynamic data on the global daily number of confirmed cases and deaths [2]. This dashboard, based on dynamic data, facilitates an understanding of the global situation of the pandemic. According to the analysis of hospitalization and, consequently, death from COVID-19 from the global archive, patients in East Asia, including Taiwan, are at a lower risk than those in America, Europe, Middle Asia, and South Asia owing to the absence of inherited genomes from Neanderthals [3]. Moreover, parts of the endemic coronaviruses that cause seasonal colds can relieve severe illness resulting from COVID-19 [4]. In addition, people in parts of East Asian countries, including Japan and Taiwan, have been accustomed to wearing masks since the 2003 SARS-CoV pandemic. Wearing a mask was also an important factor for fewer hospitalizations and better immunization after infection with COVID-19 [5]. Research on the benefit of wearing face masks during the pandemic in the US has been conducted. Evidence that the spread of COVID-19 can be mitigated with the use of face masks in public has been provided by [6]. Furthermore, reducing the spread and hospitalization risk of COVID-19 can be realized using a good management system, including the control of medical resources and regulation of the supply of face masks.

The COVID-19 pandemic has greatly impacted health care systems worldwide. According to Biryukov et al. (2020), SARS-CoV-2 can exist in an environment with a temperature of 35 °C and relative humidity of 60% for 2 h or a temperature of 24 °C and relative humidity of 20% for 15 h [8]. A dry environment with a lower temperature is associated with a higher risk of virus spread. A map system is used to facilitate the analysis of the temporal and spatial environments of COVID-19 cases, including the relationship between weather indicators and confirmed cases in New York [9] and major cities in China [10,11]. Qi et al. (2020) analyzed the relationship between infection and weather conditions among 30 provinces in China. They found a positive relationship between environments with lower temperatures and relative humidity and a larger number of infected cases [12].

Moreover, restrictions on international travel for disease prevention have led to a significant decline in global economic activity [13,14]. Maps and tracking systems were being used globally, as geographic distribution was one of the keys to understanding and fighting against this pandemic [15,16,17]. The indicators derived from the data of electricity usage and the changed labor market caused by the COVID-19 outbreak were applied to monitor economic activity [18]. In China, the results of temporal and spatial distribution analysis for population emigration were utilized as the reference for the preparation of new outbreaks [19]. Researchers in the United States and India tried to use the data of GIS-based spatial variability and spatial distribution for providing useful insights and advice to decision-makers [20,21].

Irwansyah et al. (2020) argued that a COVID-19 dashboard should be developed for the Indonesia archipelago, which could help county/city governments develop effective COVID-19 disease management and control strategies [22]. Besides tabulating the confirmed cases of COVID-19, *The Economist* (2020) started to display the distribution of vaccinations for each country, which allows users to rapidly look up information on vaccinations in various countries [23].

The disaster intelligence website was created by our team, who are members of the National Science and Technology Center for Disaster Reduction, mainly focusing on natural disasters such as typhoons, heavy rainfall, and earthquakes. The Center is used for the rapid and efficient management of disasters and has been made available to the public servants of county/city government and the Emergency Operation Center since 2010 [24]. Moreover, a tool for pandemic management was also required by the cross-organizational cooperation of personnel with varied expertise in order to ensure the compilation of timely data during the pandemic. Specified data during the period of the pandemic, including the dynamic number of confirmed cases, medical institutions, mask inventories, and human movement, have been collected and stored in an archive for creating a pandemic intelligence system that is accessible for county/city governments. The capability for real-time analysis in the system was also necessary to understand whether the strategies of disease prevention needed to be adjusted for pandemic control and management.

The pandemic intelligence system was created as a subsystem of the disaster intelligence website, going online quickly in early March 2020. This article presents the structure of the pandemic intelligence system in Section 2. The contents of the system are introduced in Section 3. In particular, it focuses on the function of map-searching with special tasks, from the point of view of the public servants in the county/city government. The 4-day weekend for Tomb-Sweeping Day in April 2020 is used as a case study for the analysis of dynamic mobile communications data in Section 4. The discussion and conclusions are presented in Section 5 and Section 6, respectively. 

## 2. Research Method

In this study, cross-organizational data were used to build a pandemic intelligence system for Taiwan’s county/city governments, intended to provide assistance in monitoring and controlling the pandemic. Traditional, all-real-time pandemic information, including hospital information and patients’ details, needed to be collected and managed by them via different platforms. The designed system then focused on its use among the public servants of the county/city government via a single platform with integrated comprehensive data. In this system, academic users can provide suggestions for management to the county/city governments via an analysis of the information from the system. The system can also provide a comparison of situations between each county/city government and the central government, for policy adjustment.

**Figure 2:**
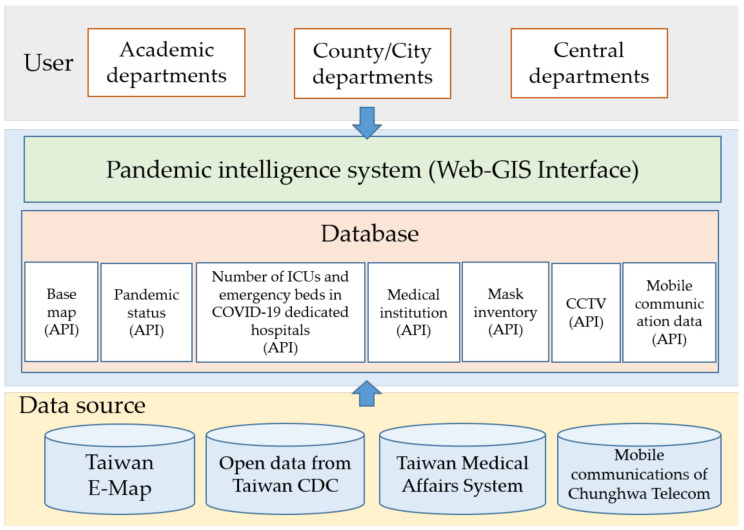
Structure of the pandemic intelligence system.

Therefore, the platform displayed pandemic information by county and city, and the information covered electronic maps, public and dynamic health insurance data, the number of negative-pressure rooms and emergency beds retrieved from the Taiwan Medical Affairs System, and the estimated population from mobile communications data related to the pandemic. The schematic layout of the pandemic intelligence system is shown in Figure 2. The introduced resources for the system included the open data of the Taiwan Centers for Disease Control, real-time video streams from the county/city governments and the transportation agency, Taiwan Medical Affairs System, and the mobile communications archive of Chunghwa Telecom. An application programming interface (API) was created and used for scheduled data access and grabbing. The grabbed data were introduced into the archive built in a Microsoft SQL Server, including the hospital capacity, the number of available face masks in stock, the real-time number of new cases, and mobile communications. The received spatial information, including digital maps and coordinates of hospitals, pharmacies, and real-time cameras, was stored in the ESRI Geodatabase Spatial Database Engine (ArcSDE).

The information interfaced by this pandemic intelligence system includes the following.

### 2.1. Geographic Map Data

The geographic map data were based on the Taiwan e-Map created by the National Land Surveying and Mapping Center. The electronic maps included layers for administrative boundaries, roads, railways, water systems, major landmarks, and buildings.

### 2.2. Daily Pandemic Updates

The pandemic data in the system were collected from the open data released from the Taiwan Centers for Disease Control. The data were updated once daily and included the number of confirmed cases per day and their sex, age, county or city of residence, the number of individuals released from isolation, the number of deaths, the number of individuals awaiting test results, and the number of individuals testing negative.

### 2.3. Hospital Resources

Dynamic data on resources in 212 COVID-19-dedicated hospitals, provided in the Medical Affairs System, were compiled in the system and updated hourly. The data included basic information, such as the hospital name, address, telephone number, and level; data on equipment, such as bronchoscopes and hyperbaric oxygen; and the number of intensive care unit (ICU) beds, negative-pressure room beds, emergency observation beds, and artificial ventilation apparatuses, as well as real-time information on whether the hospitals had sufficient emergency physicians and beds and whether they could accept additional emergency patients.

### 2.4. Mask Inventories

The health insurance data covered inventory levels in 5210 mask sales outlets around Taiwan, comprising 4871 pharmacies, 325 public health centers, 12 service centers, and 2 special hospitals, as well as the address, telephone number, and service hours of each sales outlet. Dynamic inventory data on adult and child masks were updated every 10 min. Data were acquired from the Taiwan Centers for Disease Control Open Data Platform.

### 2.5. Use of Mobile Communication Data

Chunghwa Telecom has approximately 10.649 million users in Taiwan, representing 36.45% of the total users of 29.208 million. Grid mobile communications data provided by Chunghwa Telecom were used to estimate the total number of individuals in each grid. The mobile communications data were updated every 10 min, and the grid resolution was 500 m × 500 m. Chunghwa Telecom data on mobile communications are available in 20,000 grids around Taiwan. Dynamic data on the total number of mobile communications in each grid were calculated and provided by Chunghwa Telecom. Each number represents the total number of mobile communications in the corresponding grid in the previous 10 min.

### 2.6. Real-Time Closed-Circuit Television (CCTV) Images

CCTV footage of traditional markets, tourist attractions, provincial expressways, and national freeways was provided by the county/city governments, Directorate General of Highways, and Freeway Bureau. The footage was updated based on the needs of the department that maintains and monitors the camera. Some footage was streamed in real time, while some was captured at fixed times (e.g., every 5 s).

The location and/or position were given for the data mentioned above and linked with the archive to keep the archive up to date in real time. The study was conducted using a web-based geographic information system (Web-GIS) as the design interface. Computers, tablets, and phones can connect to the system via the Internet. Users can navigate the interface by clicking on the thematic tabs of their choices, which will direct them to the corresponding themes. They can rapidly look up the status of medical resources, confirmed cases, and the supply and sales of medical masks, as well as real-time information on human traffic flow in major locations.

## 3. Results

The dynamic data compiled in the completed pandemic intelligence system are provided for real-time display on the Disasters Intelligence website.

### 3.1. Dynamic Data on the Pandemic Spread

**Figure 3:**
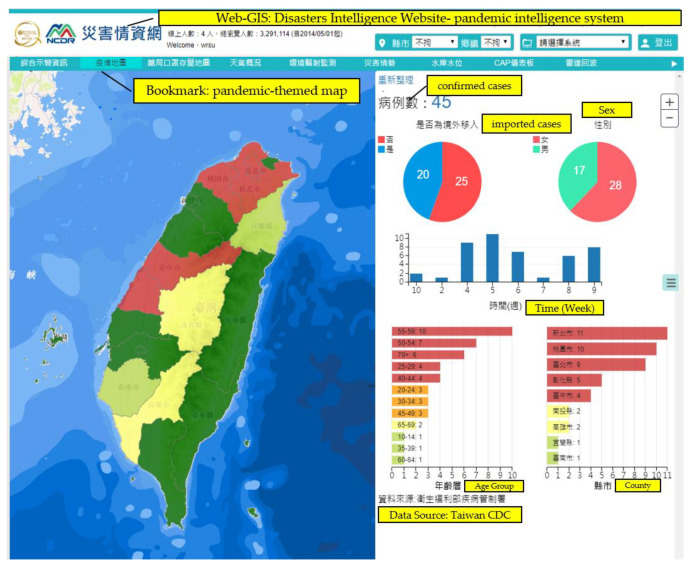
Pandemic intelligence system—dynamic information on the pandemic spread.

Daily updates on confirmed COVID-19 cases were built into the system for spatial information, based on the Taiwan e-Map. Users can use the system to gain insight into the spatial distribution of the spread of the pandemic. The pandemic-themed map (Figure 3) allows users to rapidly look up information on confirmed cases within the selected spatial range, such as age range, sex, and whether they are local or imported cases. This allows them to obtain specific local information on the pandemic cases. Figure 3 shows the situation on 3 September 2020. The total number of confirmed cases was 45, including 20 imported cases and 25 locally acquired cases, which can be identified using the map and/or histograms. For the geographic distribution, the confirmed cases were located in New Taipei City, Taipei City, and Taoyuan City in north Taiwan, and Taichung City and Changhua County in middle Taiwan. For the age group of the confirmed cases, 55–59 years old was the main age distribution, followed by 50–54 years old and over 70 years old. Regarding sex, the proportion of affected female patients was larger than that of affected male patients. 

### 3.2. Dynamic Distribution of Hospital Resources

**Figure 4:**
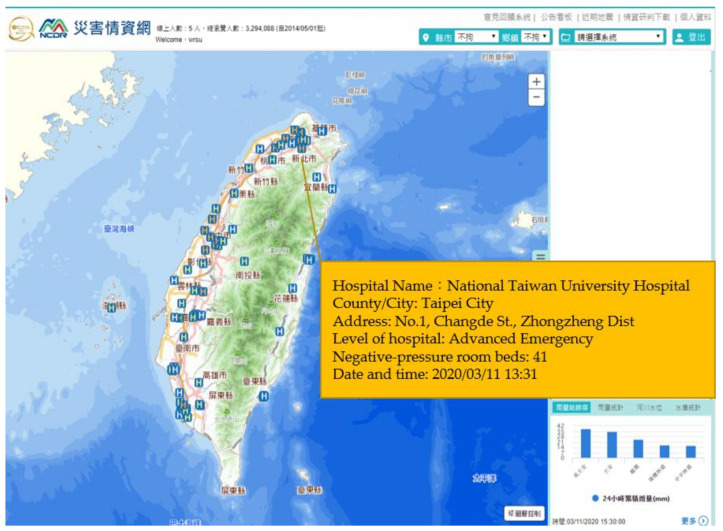
Pandemic intelligence system—dynamic distribution of hospital resources.

The dynamic distribution of hospital resources (Figure 4) included the basic information of dedicated hospitals in each county or city and their attributes, the number of emergency observation beds, the number of negative-pressure rooms, and the current usage status of the beds and rooms in each hospital. This allows users to look up real-time information on each hospital and time-series data on the number of beds, to gain insight into changes in its medical resource use over time and, thus, ensure the precise allocation of hospital resources. By understanding the capacity of hospital resources, users will hopefully be able to select hospitals with more resources to stabilize the health care capacity in Taiwan. Figure 4 shows the situation on 11 March 2020 of the National Taiwan University Hospital, giving an example of the distribution of a medical resource. Forty-one negative-pressure room beds were still available for the confirmed cases.

### 3.3. Dynamic Information on Mask Inventories

**Figure 5:**
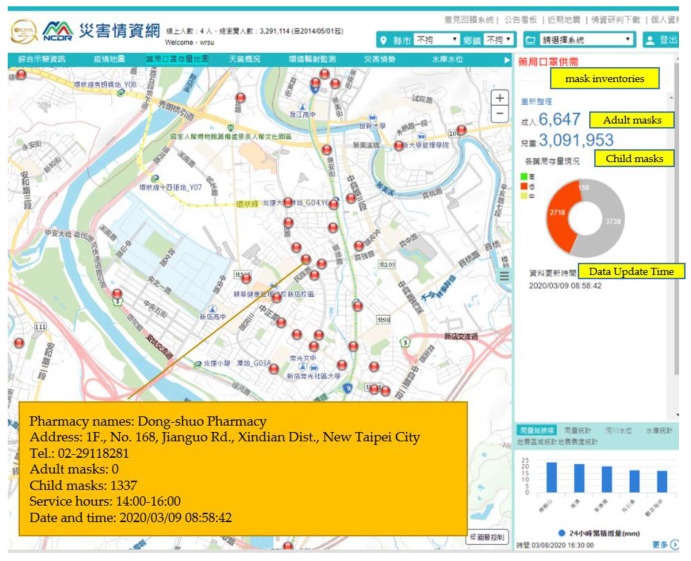
Pandemic intelligence system—dynamic data on mask inventories.

At the beginning of the COVID-19 pandemic in 2020, the Central Epidemic Command Center (CECC) urged the public to wear masks, wash their hands regularly, and maintain social distancing. Since mask use was made mandatory for public transportation, worry over mask shortages led to panic-buying. Therefore, the Taiwanese government developed a name-based system for selling masks, which regulated the number of masks an individual could buy each day. A total of 5210 pharmacies were entrusted with selling masks. The information on each pharmacy would include service hours, location, telephone, and inventory levels of adults’ and children’s masks, updated every 10 min. Figure 5 shows the situation on 9 March 2020. The selected Dong-shuo Pharmacy had 0 adult masks and 1337 child masks in stock. The total number of available masks in Taiwan on this date is presented on the left side bar. 

### 3.4. Dynamic Information on Crowds

**Figure 6:**
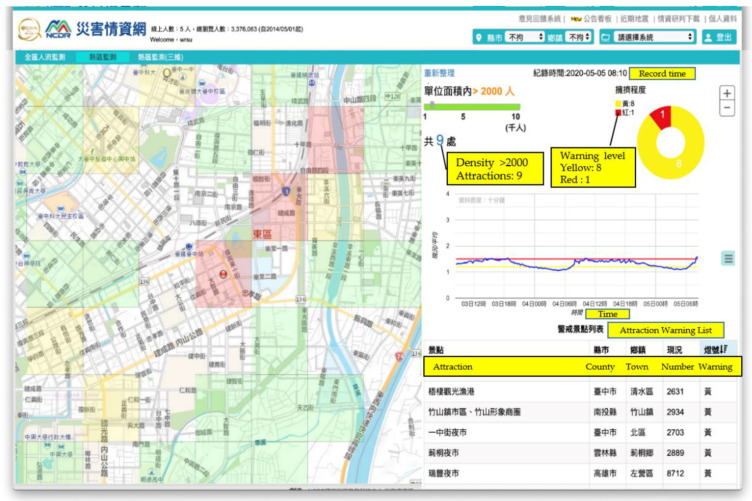
Pandemic intelligence system—dynamic information on crowds and automated alerts.

During the COVID-19 pandemic, Taiwan initially did not implement lockdowns or school closures, and there was no significant community spread at that time. Under these conditions, encouraging the public to wash their hands, wear masks, and maintain social distancing was the highlight of Taiwan’s COVID-19 prevention policy. The prevalence of phone use in Taiwan is close to 100%; thus, mobile communications data can provide clear insight into crowding in various areas, allowing the issuance of timely alert reminders to avoid busy areas. This system calculates the total number of individuals with mobile communications within the Chunghwa Telecom grid. The numbers represent the total number of users of mobile communications that stayed within the 500 m × 500 m grid during the previous 10 min. The system can analyze changes in the dynamic data on mobile communications to determine the presence of crowds and enable real-time analyses and alerts (Figure 6). The base values are each grid’s mean total for each time interval in March 2020; a grid will turn yellow when the number exceeds 1.2 times the base value and red when the number exceeds 1.5 times the base value. Users can select a grid to see human traffic-flow changes in the previous 72 h. Figure 6 shows the situation at 8:10 a.m. on 5 May 2020. There were nine grids with a population of over 2000. One of them was up by an amount over 1.5 times that of the normal period. The system can let users check the population of an area in real time, as well as the time series for the previous 3 days. The cycle change or aggregate peak can be identified easily via the system. 

According to the data released from the Taiwan Centers for Disease Control, the local COVID-19 confirmed cases were down to zero from 13 April to the end of 2020. During the period of time from March to April 2020, the most used map layer is that regarding face masks, the usage of which reaches 819. The crowd distribution map is 729. However, from March to December 2020, the most-used layer was the “dynamic information on crowds and automated alerts”. The number of times of usage can reach 1823. The layer of “dynamic data on mask inventories” was the second most-used layer, and the number of uses can reach 1573 times. The layers of “dynamic information on pandemic spread” and “dynamic distribution of hospital resources” are 308 and 100, respectively. 

## 4. Analysis of Dynamic Mobile Communications Data

**Figure 7:**
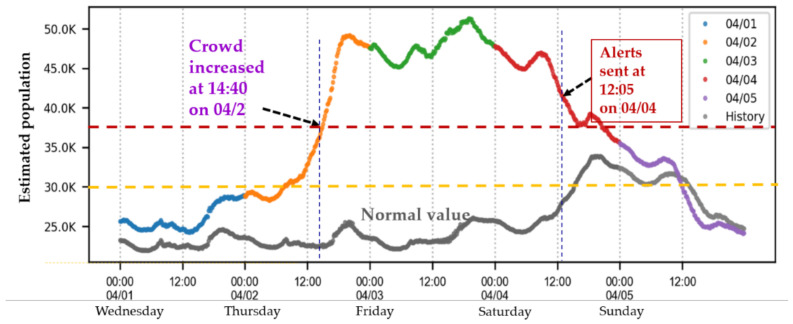
Crowd distribution in Hengchun Township from 1 to 5 April 2020, based on mobile communications.

In Taiwan, April always begins with a 4-day weekend for Tomb-Sweeping Day. In 2020, the 4-day weekend fell from 2 to 5 April. The number of confirmed COVID-19 cases was on the rise, from single digits to 15–20 at the end of March 2020, although most of the confirmed cases were imported cases. Mobile data from the Chunghwa Telecom grids were analyzed to estimate the number of individuals traveling to the most popular tourist destination in Pingtung County, Hengchun Township, during the long weekend, to identify whether large crowds were present at tourist attractions. The estimated number of individuals in Hengchun Township over the 4-day weekend, based on mobile communications, is depicted in Figure 7. The normal value was the average number of the population in the span of a week in 2019, based on the Chunghwa Telecom grids for the Hengchun Township area. Excluding long weekends and flexible working days, a total of 35 weeks were counted to obtain the average of the sub-span. The normal values were quite flat during the weekdays but increased from Saturday afternoon. After Sunday afternoon, lower numbers were observed. Figure 7 indicates a gradual increase in the number of individuals in Hengchun Township, beginning on the afternoon of 2 April, and the peak was maintained throughout 3 April (approximately twice the normal number of individuals) before decreasing on 4 April.

**Figure 8:**
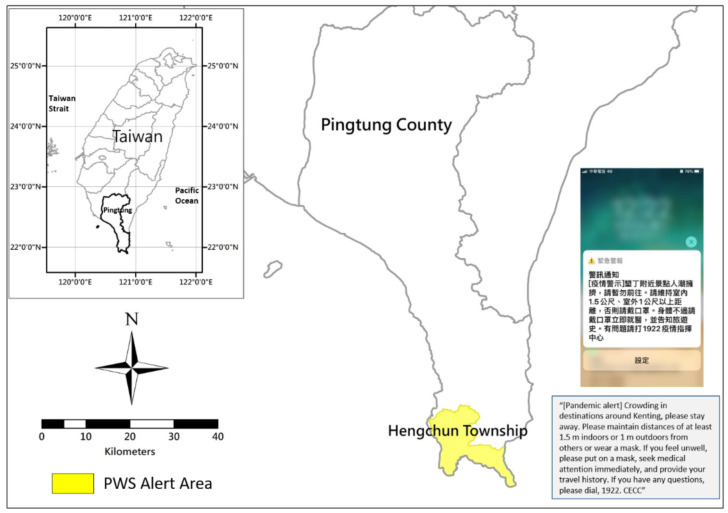
PWS message sent on 4 April by the CECC.

Notably, at 12:05 p.m. on 4 April, the CECC sent out a push notification on the public warning system (PWS) warning the public to avoid crowded destinations. The message, in English translation, reads, “(Pandemic alert) Crowding in destinations around Kenting, please stay away. Please maintain distances of at least 1.5 m indoors or 1 m outdoors from others or wear a mask. If you feel unwell, please put on a mask, seek medical attention immediately, and provide your travel history. If you have any questions, please dial 1922. CECC” (Figure 8).

**Figure 9:**
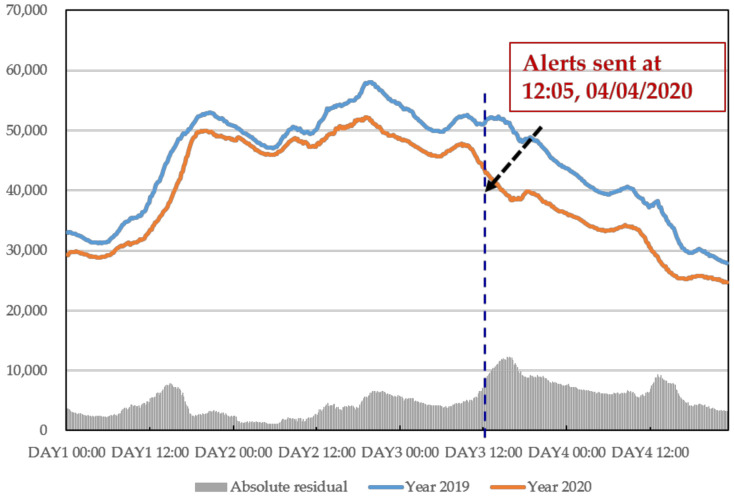
Crowd distributions in Hengchun Township over 4-day weekends in 2019 and 2020.

Comparing data in Hengchun Township on the 4-day weekends in 2019 (4 to 7 April) and in 2020 (2 to 5 April), the PWS message led to a significant decrease in crowd size by 2020 (Figure 9). Based on the data on the consecutive 4-day and 3-night holidays in 2019, the crowds began dissipating at approximately 5:00 p.m. on the third evening. In 2020, after the PWS alert was sent out by the CECC at noon on the third day, the crowd size decreased rapidly. The absolute residual was the difference in the estimated population between 2019 and 2020. The absolute residual value was in the range of between 1086 and 12,329. The value over 10,000 happened from 13:00 to 16:50 on DAY 3.

The specified 4 days were investigated and analyzed using the estimated population of the Hengchun Township, and the results are presented in Table 1. The estimated population in 2020 was less than it was in 2019 for each day. A high correlation between 2019 and 2020 for Day 1, Day 2, and Day 4 was observed. Both the Pearson correlation and R-square were over 0.9. Particularly for Day 1, the Pearson correlation reached up to 0.98. However, there was no high correlation observed for Day 3 between 2019 and 2020; only an R-square of 0.6 was obtained. These values should be consistent with the existing factors affecting changes in crowd distribution. The statistical analysis of the estimated population from mobile communications data indicated that the public adjusted their pandemic prevention behavior after receiving a government advisory text and that these alerts were effective forms of pandemic management. Moreover, mass media reported people driving away, as well as the check-out status of visitors from the resorts in Kenting in Hengchun Township after the warning dissemination [25]. The accelerated dispersion was demonstrated as evidence after the alert sent by the CECC.

## 5. Discussion

The scope of the COVID-19 pandemic in 2020 was quite broad, affecting the inventories of emergency medical resources, advocacies for social distancing, and purchases of masks. This study aimed to provide integrated information for the public servants of county/city government via Web-GIS. The findings can serve as a reference point and strategy for pandemic management. Based on the information given, the most-used layer in the archive was that of population monitoring for the cluster, followed by the number of face masks in stock and the geographical distribution of infected cases in the pandemic-themed map. The layer on medical resources was not frequently used. In Taiwan, wearing face masks and avoiding aggregation demonstrate the disseminating of information as the main government policy for controlling the epidemic. This is the main reason why public servants of county/city governments always focus on the number of face masks in stock and the monitoring of populations. In Taiwan, the race and climate conditions were not significantly different. The confirmed cases were mainly distributed in the central business district of Taipei in the north and Taichung city in the south. Currently, there are insufficient data to prove the relationship between the pandemic and climatic conditions in Taiwan. Moreover, because of the lower number of confirmed cases and deaths in Taiwan, the lower number of requests and the monitoring of medical resources from the system are represented.

Data on crowds are derived from 500 m × 500 m grid-based mobile communications totals, which could lead to excessive automated alerts in metropolitan or mixed residential and commercial areas. If the area of scenic spots is used as the target of the monitor, the greatest benefit of the application of the system is achieved. Compared with the estimated population in 2019, the estimated population in 2020 shows a significant decline in the total number of tourists in the Hengchun Township. The impact of the pandemic on tourism in Taiwan may need extended information-gathering for further study. In this study, this system has already been successfully used in the Hengchun Township in April 2020. In addition, based on mobile communications, if a warning regarding social distancing is provided in situ in a crowded area, individuals will make decisions to change their itinerary, as individuals agree to follow the rule when staying in a public crowded area [26]. Such disaster-avoidance behavior by the public has made Taiwan’s present success in pandemic prevention possible, which has earned significant international attention.

## 6. Conclusions

Based on the current existing experiences on disaster management, including the integration of fundamental data, real-time data, warning information, and application system for the pandemic, our team was built rapidly; the system provides useful real-time dynamic data and spatial information with a GIS-based interface for county/city governments to control the pandemic. During the period of time from March 2020 to the end of that year, the most-used layer was the crowd distribution information in the thematic map of the pandemic; the number of times it was used reached 1823. The layer regarding face masks in stock was the second most-used layer, up to 1573 times. The most important task for pandemic prevention for the county/city governments is to avoid clusters of people. The local COVID-19 confirmed case rate was zero from 13 April to the end of 2020. Therefore, the 4-day weekend for Tomb-Sweeping Day was the most significant example in the study period. However, it is not easy to justify the use of this system, and the specific relationship with COVID-19 incidence is a limitation of this investigation.

Although the benefits of the system have been highlighted in the case of issuing overcrowding warnings for scenic spots, the county/city governments wanted to pay more attention to traditional markets because of the high-density population in these and commercial and residential mixed areas. Thus, there will be more restrictions on monitoring crowd distribution in this context. In the future, the size of grids will be reduced from 500 m × 500 m to 250 m × 250 m, or less than 250 m × 250 m, and resident and floating populations will be distinguished to improve the resolution as the key point of further system development.

The vaccination figures and confirmed exposure locations are also important factors because of the more serious situations observed therein. However, they are also related to privacy and this issue can make a single, integrated, and easily used platform difficult to achieve. Once established, the system can provide the rapid integration of pandemic information and dynamic data across all government departments, canvassing the needs of epidemic-prevention professionals, compiling information from different channels on confirmed COVID-19 cases, medical resources, and mask resources, and analyzing the presence of crowds based on mobile communications data. The effectiveness of pandemic prevention in Taiwan relies not only on the efforts of epidemic prevention professionals but also on the voluntary cooperation of the public.

## Figures and Tables

**Table 1:** The statistical analysis of the 2019 and 2020 Hengchun Township’s estimated population from mobile communications data on each day.

		Mean	Standard Deviation	Pearson Correlation	R-Square	Standard Error
Day 1	Year 2019	41,016.25	8401.18	0.9809	0.9622	1638.94
	Year 2020	37,194.01	8226.57			
Day 2	Year 2019	52,028.64	3378.98	0.9366	0.8773	1187.89
	Year 2020	48,765.62	1763.57			
Day 3	Year 2019	50,096.36	2547.64	0.7807	0.6095	1597.56
	Year 2020	42,943.91	4086.25			
Day 4	Year 2019	36,179.27	4989.93	0.9638	0.9290	1334.70
	Year 2020	30,173.23	4075.5			

## Data Availability

Publicly available datasets were analyzed in the present study. The data can be found here: https://data.cdc.gov.tw/dataset/aagsdctable-day-19cov (accessed on 10 September 2021); https://data.gov.tw/dataset/39283 (accessed on 10 September 2021); https://data.gov.tw/dataset/116285 (accessed on 10 September 2021).
